# CRBPDL: Identification of circRNA-RBP interaction sites using an ensemble neural network approach

**DOI:** 10.1371/journal.pcbi.1009798

**Published:** 2022-01-20

**Authors:** Mengting Niu, Quan Zou, Chen Lin

**Affiliations:** 1 Institute of Fundamental and Frontier Sciences, University of Electronic Science and Technology of China, Chengdu, China; 2 Yangtze Delta Region Institute (Quzhou), University of Electronic Science and Technology of China, Quzhou, Zhejiang, China; 3 School of Informatics, Xiamen University, Xiamen, China; University of North Texas, UNITED STATES

## Abstract

Circular RNAs (circRNAs) are non-coding RNAs with a special circular structure produced formed by the reverse splicing mechanism. Increasing evidence shows that circular RNAs can directly bind to RNA-binding proteins (RBP) and play an important role in a variety of biological activities. The interactions between circRNAs and RBPs are key to comprehending the mechanism of posttranscriptional regulation. Accurately identifying binding sites is very useful for analyzing interactions. In past research, some predictors on the basis of machine learning (ML) have been presented, but prediction accuracy still needs to be ameliorated. Therefore, we present a novel calculation model, CRBPDL, which uses an Adaboost integrated deep hierarchical network to identify the binding sites of circular RNA-RBP. CRBPDL combines five different feature encoding schemes to encode the original RNA sequence, uses deep multiscale residual networks (MSRN) and bidirectional gating recurrent units (BiGRUs) to effectively learn high-level feature representations, it is sufficient to extract local and global context information at the same time. Additionally, a self-attention mechanism is employed to train the robustness of the CRBPDL. Ultimately, the Adaboost algorithm is applied to integrate deep learning (DL) model to improve prediction performance and reliability of the model. To verify the usefulness of CRBPDL, we compared the efficiency with state-of-the-art methods on 37 circular RNA data sets and 31 linear RNA data sets. Moreover, results display that CRBPDL is capable of performing universal, reliable, and robust. The code and data sets are obtainable at https://github.com/nmt315320/CRBPDL.git.

## Author summary

More and more evidences show that circular RNA can directly bind to proteins and participate in countless different biological processes. The calculation method can quickly and accurately predict the binding site of circular RNA and RBP. In order to identify the interaction of circRNA with 37 different types of circRNA binding proteins, we developed an integrated deep learning network based on hierarchical network, called CRBPDL. It can effectively learn high-level feature representations. The performance of the model was verified through comparative experiments of different feature extraction algorithms, different deep learning models and classifier models. Moreover, the CRBPDL model was applied to 31 linear RNAs, and the effectiveness of our method was proved by comparison with the results of current excellent algorithms. It is expected that the CRBPDL model can effectively predict the binding site of circular RNA-RBP and provide reliable candidates for further biological experiments.

This is a *PLOS Computational Biology* Methods paper.

## Introduction

Circular RNA (circRNA) is a special circular endogenous noncoding RNA produced by selective shearing [1,2]. It has been proven to be widely present in Drosophila, mice, the hippocampus and human cells and tissues [3,4]. Although the RNA-binding proteins (RBP) binding sites on circular RNAs are less numerous than those on linear mRNAs, there is still strong evidence to support the interaction of RBPs with circular RNAs [5,6]. On the one hand, circRNAs can regulate RBPs in a variety of ways. CircRNAs can competitively bind to RBPs, regulate the function of RBPs, and act as sponges of RBPs, platforms for RBP assembly, and supertransporters that concentrate certain specific components [7,8]. RBP-adsorbed circRNA can be used as a regulatory factor for target gene transcription and splicing [9]. circRNA can also be used as a bait to retain RBPs in a specific intercellular space and as a scaffold to promote contact between two or more RBPs [10]. On the other hand, the influence of RBPs on circRNAs is becoming increasingly prominent. As a protein that binds to double-stranded or single-stranded RNA, RBPs are present throughout the life of RNA and mediate the maturation [11], transport [12], positioning and translation of RNA [13]. RBPs affect the entire process of the circRNA life cycle, and some RBPs are also involved in the generation of circRNAs, such as Quking (QKI), FUS, and HNRNPL. Moreover, they are involved in almost every aspect of the cyclic RNA life cycle, including generation [14], posttranscriptional regulation [15], and functional execution [16]. Some specific RBPs are tissue-specific or produced under pathological conditions, and their expression defects can cause a variety of diseases and other effects. Multiple studies have shown that the interaction between circular RNA and RBP has an important impact on cancer and other diseases and may be a disease of biomarkers [10,17–21]. Therefore, predicting the binding site of RNA and RBP can provide insight into the mechanisms underlying diseases involving RBPs and help to further explore the role of circRNA in disease pathophysiology.

As a promising method, machine learning has been used to solve various biological problems, its superiority has been proven many times, and it has gradually been used to identify the binding sites of circular RNA-RBP [22]. Matizka et al. proposed the GraphProt method, which can learn secondary structure characteristics, and used support vector machine (SVM) to predict binding sites and affinity of RBPs in all tissues [23]. Corrado et al. applied recommendation algorithm to recommend RNA targets for RNA-binding proteins based on protein domain composition and RNA predicted secondary structure features [24]. Yu et al. employed the random forest algorithm (RF) to predict specific and general RBP sites based on motif information [25]. The above machine learning models are mainly based on the structural characteristics of RNA sequences to identify the binding sites of circular RNA-RBP [26,27].

Deep learning has fulfilled remarkable accomplishment in the field of bioinformatics recently [28–30], which also includes the prediction of RNA-protein interactions The DeepBind method utilized convolutional neural network (CNN) to learn binding preference of individual RBPs and obtains better performance [31]. Pan et al. proposed iDeepE method, which uses the global CNN model to predict the binding site by studying RNA sequences [32]. In addition, they further used two separate CNNs and a long-term short-term memory network to learn the sites [33]. Pan et al. further used multilabel classification and deep learning to identify multiple RBPs that can interact with RNA [34]. Jia et al. constructed an hybrid deep neural network [35]. Zhang advanced a new stacked codon coding scheme and combined it with hybrid deep learning to complete the prediction [36]. Yang et al. constructed a multiscale neural network and predicted the binding site of circRBA-RBP based on contextual sequence information [37]. However, the feature learning network is relatively simple, and there is still potential for improvement in prediction performance.

**Fig 1:**
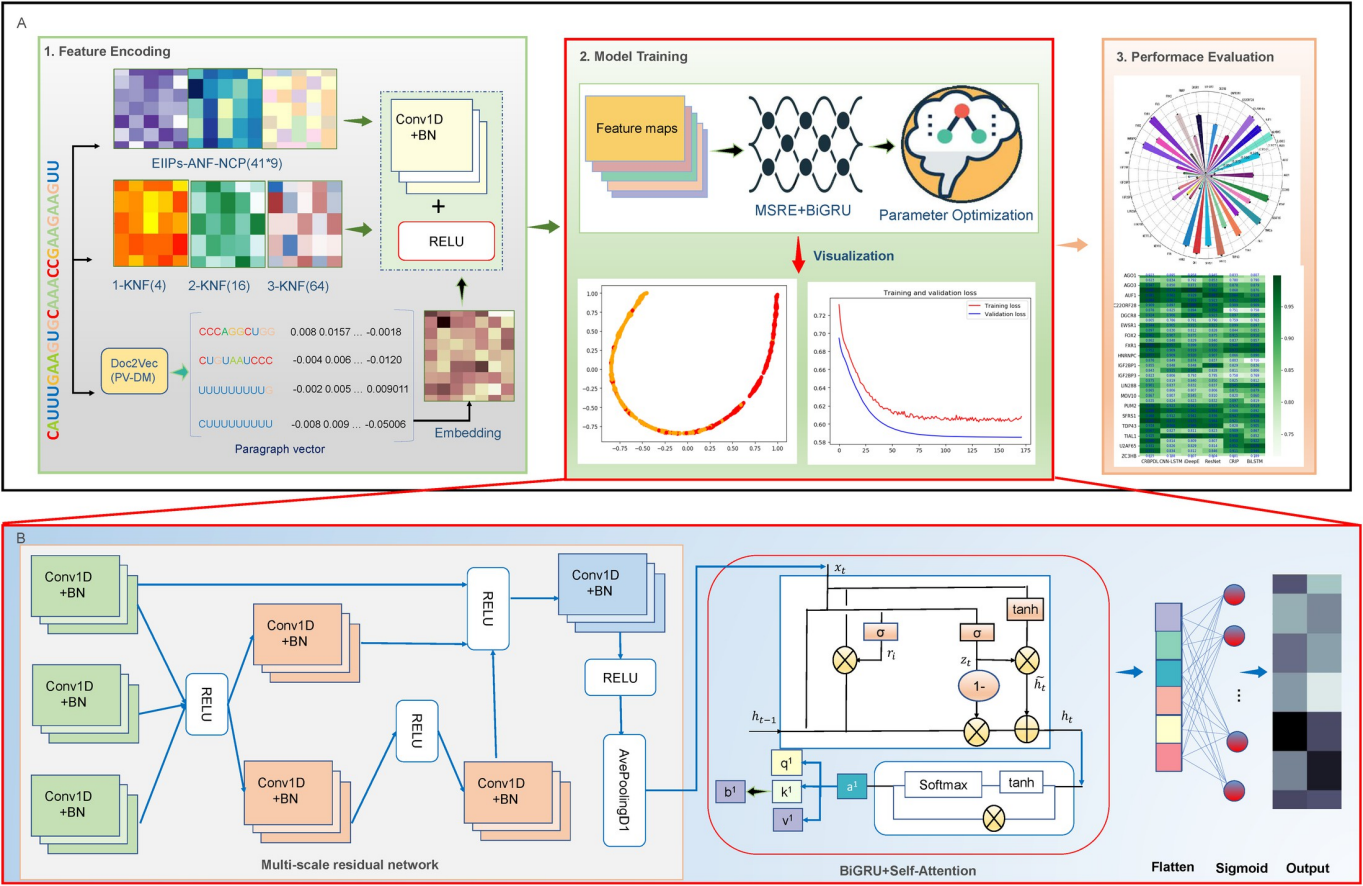
The overall framework of CRBPDL. (A) The workflow of the development and assessment process of CRBPDL. (B) The structure of the sig-CRBPDL framework, including the input layer, convolutional layers, merger layers, inception module, attention layers, fully connected layers and output layer.

In our work, we establish a novel computational predictor CRBPDL, which based on an ensemble deep network to identify circRNA-RBP interaction sites. First, we adopted 5 coding schemes to provide comprehensive feature information for model training, including k-nucleotide frequency (KNF), Doc2vec, electron-ion interaction pseudopotential (EIIP), nucleotide chemical properties (CCN) And cumulative nucleotide frequency (ANF). Due to the different distributions of feature descriptors, we first applied convolution filters to the features respectively, and then concatenated them into a feature matrix. Subsequently, to automatically extract high-order local and global context information from feature descriptors, we constructed a deep neural network architecture, which consists of a deep multi-scale residual network (ResNet) and a bidirectional gated recurrent unit with a self-attention mechanism (BiGRUs) network composition. We used deep multi-scale residual networks (MRSN) and BiGRUs to learn local and global contextual information, and can effectively represent high-level features. Then, used the self-attention mechanism to train the robustness of the model. After model training and selection, we can get the optimized deep learning model (for convenience, the deep learning model before integration is named "sig-CRBPDL"). Finally, the AdaBoost algorithm was used to integrate the deep learning model. We benchmarked CRBPDL and existing predictors on the unified circRNA dataset. The benchmark test results clearly showed the superiority of our proposed CRBPDL. In addition, CRBPDL has the potential to recognize linear RNA-RBP interaction sites. The benchmark results showed that CRBPDL also has stable performance in predicting linear RNA-RBP binding sites. The structure of the CRBPDL model is shown in Fig 1.

## Results

### Model performance under different network layers

**Fig 2:**
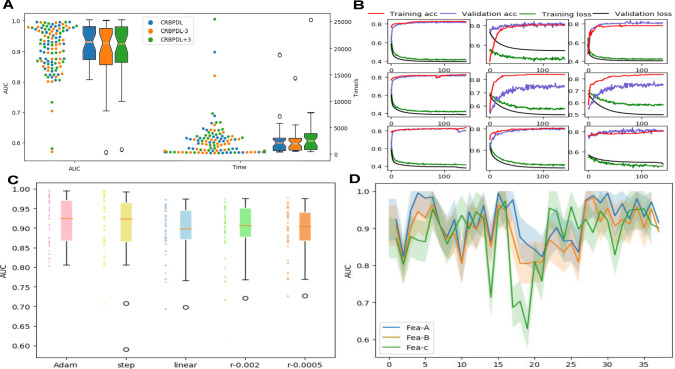
(A) Comparison of model performance between different network depths visualized by box and fiddle charts. (B) Model performance analysis under different EPOCH. (C) Comparison of model performance under different learning rate schemes. (D) Comparison of model performance under different feature coding schemes.

Network depth has great effects on the performance of deep learning models. Different network depths will lead to diverse results. A relatively shallow network will make the model perform poorly, and an overly complex network will increase the calculation of the model. This section analyzed the model performance changes under different network layers. We compared the increase and decrease: reducing one MSRB block, that is, a 3-layer convolutional network layer, and adding an MSRB block, which means adding a 3-layer convolutional neural network, respectively named CRBP-3 and CRBP+3 for convenience of description. We calculated the prediction performance of CRBPDL, CRBPDL-3 and CRBPDL+3 (AUC as an evaluation index) and running time under 37 data sets (Fig 2A).

First, it can be seen from the scatter plot that the AUC value distribution of CRBPDL is 0.9174, which is higher than that of CRBPDL-3 (0.8995), and the running time is the opposite. The AUCs of the CRBPDL and CRBPDL+3 (AUC is 0.9011) distributions are not very different, but the running times are quite different. On the other hand, by observing the distribution of the maximum, minimum, and average values in the box chart, it can be found that the midline positions of the three are similar, but the bottom positions of the CRBPDL-3 and CRBPDL+3AUC box charts are lower. The top position of the box chart for CRBPDL+3 time is higher. The prediction performance distributions of CRBPDL-3 and CRBPDL+3 were quite different, and the performance is not stable enough. In contrast, the distribution difference of CRBPDL is smaller, and the stability is better. In terms of time consumption, the performance of CRBPDL-3 is better than that of CRBPDL, but the difference is small. This proves the complexity of the network layer may impact the behavior of the network. It also shows in practical applications, when faced with the needs of different time consumption and prediction effects, both the progressive neural network and the deep neural network have research significance and value.

### Model performance under different epoch times

This section statistically analyzes the changes in the loss and ACC of the training set and the validation set during the training phase and accordingly analyzes the impact of epoch on the model performance and the convergence of the model. If the graphs of all 37 data sets are displayed, there are too many pictures. Therefore, we only randomly selected 9 results for display, and can illustrate the effects of different data sets (the loss results of the remaining 28 data sets can be seen in S1 Text). This section mainly analyzes 9 out of 37 data sets which are AGO1, AGO2, U2AF65, DGCR8, FOX2, WTAP, EIF4A3, FMRP, and ZC3H7B. The results of the 9 data sets are shown in Fig 2B.

We can see that as the epoch time increases, the train-acc and validation-acc of CRBPDL both show an upward trend, and the overall train-loss and validation-loss show a downward trend and gradually stabilize; the model gradually converges, and training results are gradually optimized. The obvious performance of overfitting is that the performance of the training set is particularly fine, but the performance of the verification set is exceptionally poor. It can be found that in the 5th and 6th data sets of the 9 data sets, the trend of the acc curve is quite different. The performance effect of train-acc is obviously better than that of validation-acc. There is an obvious overfitting phenomenon, but not in the other 7 data sets. The reason for this difference may be that the data volumes of AGO3 and WTAP (that is, the 5th and 6th data sets) are small (1,210 and 892 data points, respectively), and the learning and training process of the CRBPDL model is not sufficient. In contrast, the data volume of the other data sets is on average one hundred times greater, achieving better training results. It can also be seen that the size of the data set is very important for the performance of deep learning neural networks.

### Model performance under different learning rate

As a hyperparameter of the neural network, the learning rate can be used to improve the performance of the model. The lower the learning rate is, the slower the gradient rate. When determining the learning rate, it’s generally essential to rely on the comparison of old experience and multiple experiments. The section analyzes the effect of the learning rate. To compare the effect, we analyzed three learning rate attenuation schemes (step-based attenuation learning rate scheme, linear learning rate attenuation scheme, polynomial learning rate scheme) and two fixed learning rates (0.002, 0.0005). Fig 2C shows the comparison of the AUC of the network optimization process when uses different learning rates.

On all circRNA data sets, the Adam method achieved an average AUC value of 0.9284, which was significantly better than the 0.8926 of the linear scheme and the effect of two fixed learning rates (average AUC of 0.8167 and 0.8747, respectively). Although it is not much different from the average AUC value of 0.9273 of the step scheme, there are two abnormalities in the step. In contrast, Adam’s performance is relatively stable. By performing an experimental comparison of five case, it shows that the Adam linear learning rate plan is always better than other types of plans and has better performance. Therefore, we choose Adam as the learning rate learning plan.

### Model Performance under different feature encoding schemes

To evaluate the contribution of the feature encoding schemes (named Fea-A) in this article, under the same CRBPDL architecture, we combined the proposed feature with the coding schemes of PASSION (named Fea-B) [35] and CRIP’s [36] stacked codon coding (named Fea-C). The AUC values of the 37 data sets are shown in the line graph of Fig 2D.

First of all, by observing the trend of the line chart, we can find that the AUC value of Fea-A is higher than that of Fea-B and Fea-C on multiple data. In addition, our method Fea-A obtains an average AUC value of 0.9201, which is not only upper than the 0.8928 of the Fea-B, but also superior than the 0.8792 of the Fea-C. For one thing, Fea-B uses 6 hand-designed features, and it is possible that a single hand-designed function is not suitable for advanced network architectures. For another thing, Fea-C is an improvement of one-hot encoding, which only uses feature type references in the pseudo-translation process. This may cause CRBPDL to fail to fully understand enough information in the circRNA-RBP interaction. Furthermore, the experimental results prove the validity of our feature encoding schemes.

**Fig 3:**
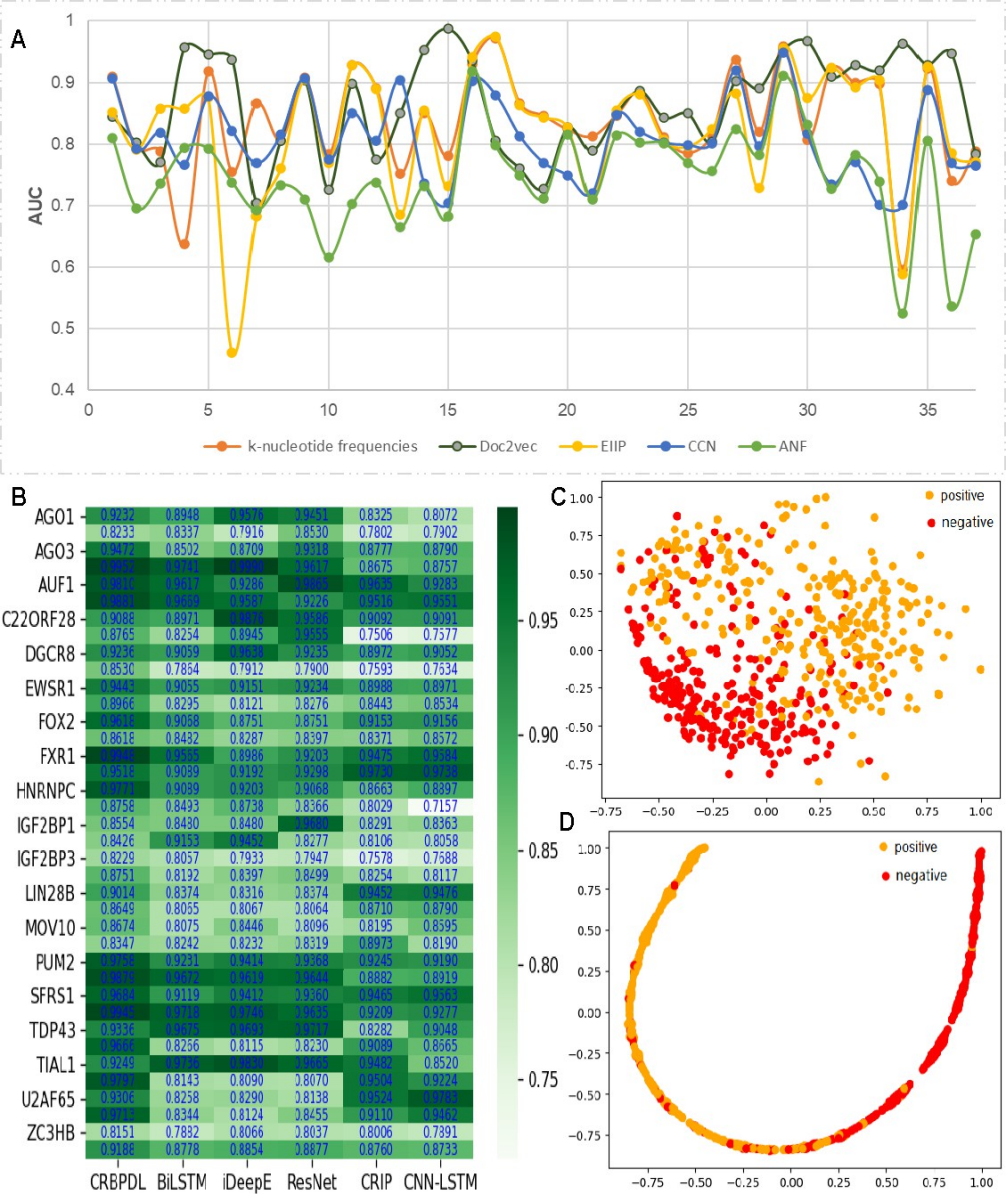
(A) Performance comparison of five feature codes.(B) Heat maps of different network model performance under 37 data sets.(C) T -SNE scatter plot with original feature coding. (D) T-SNE Scatter Diagram of Deep Feature after Deep Convolutional Network.

In addition, we analyzed the different performance of the five feature encodes, and the results were shown in Fig 3A. It can be found that on 37 data sets, the Doc2vec coding scheme is relatively better than the other four. It shows that the global text characteristics of RBP binding sites are relatively obvious. Our word vector model seems to have learned the subtle sequence context from semantics, thereby improving the recognition performance. For circRNA data sets, the experimental results show that the self-learning word vector encoding scheme proposed in this paper has a good application prospect. Based on the word vectors obtained, whether the conservative motifs of the binding sites can be analyzed.

### Performance of neural network structures with different depths

To prove the effectiveness of our proposed CRBPDL, we input our features into different CNN to compare the prediction performance of different neural networks.We compared its performance with 5 structures: CNN-LSTM, iDeepE [32], ResNet [38], CRIP-RNN [36], and CNN-BiLSTM. CNN-LSTM includes two bidirectional LSTM layers and two fully connected layers; iDeepE combines output features of the global network and local neural network, and two layers of local multichannel neural networks (convolution, ReLU and max pooling) express high-level features and then input the feature map into two fully connected layers; ResNet uses a 21-layer local multichannel network, inserts a shortcut connection between the two networks, and makes the network into a corresponding residual network; CRIP uses two layers, a CNN that extracts high-level features and a RNN that acquires the long-term dependence of sequence; and CNN-BiLSTM uses bi-directional long-short term Memory to integrate data, including two bidirectional LSTM and two fully connected layers. These network structures can be built with reference to the literature or built by themselves, and the parameters of each model have the same parameters as CRBPDL. The experimental result is displayed in Fig 3B.

As shown in Fig 3B, we find that the average AUCs of all circular RNA data sets are 0.9174, 0.8778, 0.8854, 0.8877, 0.8760, 0.8733, 0.9148, and 0.9201. When the feature codes of this article are input into different neural networks, the results obtained are different. The difference is based on whether the network structure expresses high-level features accurately. Obviously, the CRBPDL model can learn more valuable sequence information for the identification of circRNA-RBP interaction sites.

**Fig 4:**
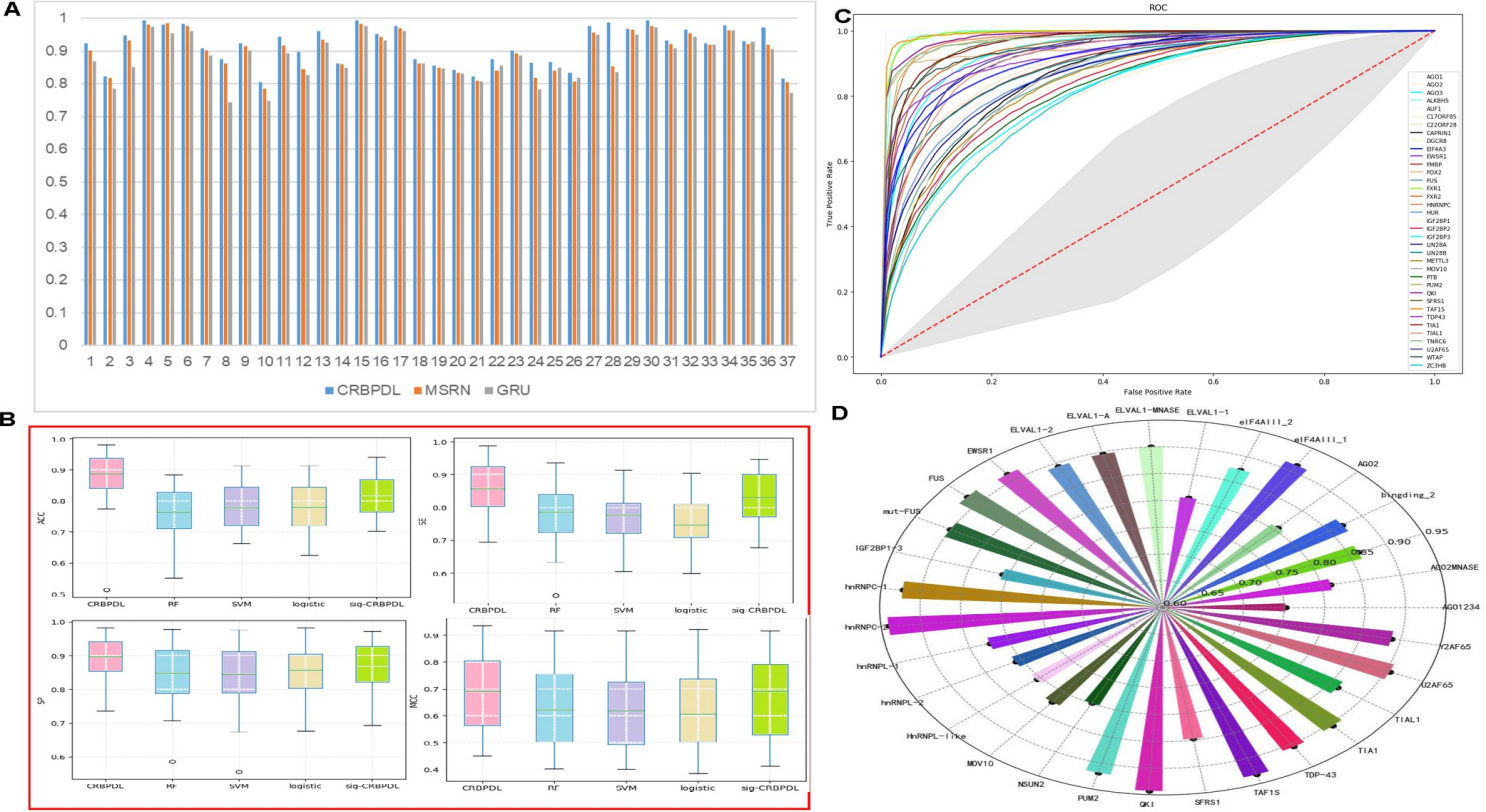
(A) Performance comparison of MSRN and BiGRU. (B) The performance comparison between CRBPDL integrated model and various classification algorithms (C) ROC curves of 37 datasets under the integration model (D) Radar chart of ACC indicators of CRBPDL model under 31 lncRNA datasets.

To demonstrate how CRBPDL learns efficient feature representation, we take the “WATP” data set as an example, and use t-SNE graphs to visualize feature representation. Both dimensions automatically learn CRBPDL. The original features are shown in Fig 3C. We can find that it is challenging to visually distinguish two categories with primitive characteristics. In addition, the second level of full connectivity after feature representation (Fig 3D) can be used to better identify and separate positive and negative examples. Graphical display shows that CRBPDL can effectively learn excellent feature representations. Moreover, we further analyzed the different performance of MSRN and BiGRU, as shown in Fig 4A. It can be found that although the difference between the two is relatively small, the effect of MSRN is significantly better than that of BiGRU, indicating that in the CRBPDL model, the contribution of MSRN is greater.

### Performance evaluation of the integration model

In this section, the CRBPDL model and the sig-CRBPDL, RF, SVM, and logistics models and four other machine learning methods are compared. Among them, the evaluation indicators include ACC, SE, SP, and MCC. The parameters of the RF [39,40], SVM, and logistics algorithms are experimented with the default parameters, and the results (ACC, SE, SP, MCC) are shown in Fig 4B. We find that the value of our proposed integrated deep network model CRBPDL on ACC, SE, SP and MCC is significantly higher than the experimental results of RF, SVM and logistics, and the average values of SE, SP, MCC, ACC are 0.8548, 0.7796, 0.6897, and 0.8739. Compared with the single deep network model sig-CRBPDL, there is also a certain level of improvement, indicating that the integrated deep learning model CRBPDL improves the prediction performance of circRNA-RBP interaction sites.

### Prediction performance of CRBPDL on 37 circRNA data sets

In this part, the prediction performance of CRBPDL, iCircRBP-DHN [37] and CRIP [36], PASSION [35], CSCRSites [41] and CircSLNN [42] and five other existing calculation methods are measured by AUC. CSCRSites was based on multiple convolutional thermal coding layers to identify cancer-specific RBP binding sites on circular RNAs. In contrast, CircSLNN used a sequence tagging network to recognize the interaction site. In terms of fairness, the six methods were tested on a unified benchmark data set, using the same sequence similarity threshold. In addition, the same setting environment is used as the model iCircRBP-DHN. The AUC results and average values of other comparative experiments are shown in Table 1, obtained directly from the literature (the maximum value was shown in bold) [37].

**Table 1:** Comparison of prediction performance under different classification models on 37 circRNA datasets.

dataset	CRBPDL	iCircRBP-DHN	PASSION	CRIP	CSCRites	CircSLNN
AGO1	**0.9232**	0.898±0.003	0.909±0.003	0.905±0.002	0851±0.002	0.844±0.002
AGO2	**0.8233**	0.797±0.004	0.822±0.003	0.811±0.001	0.755±0.127	0.715±0.013
AGO3	**0.9472**	0.920±0.016	0.909±0.008	0.895±0.002	0.820±0.004	0.860±0.006
ALKBH5	**0.9952**	0.979±0.004	0.752±0.030	0.721±0.008	0.798±0.009	0.583±0.014
AUF1	0.9810	**0.985±0.002**	0.979±0.003	0.980±0.000	0.940±0.001	0.971±0.001
C17ORF85	**0.9881**	0.987±0.002	0.860±0.021	0.813±0.007	0.815±0.012	0.721±0.010
C22ORF28	0.9088	**0.913±0.004**	0.894±0.008	0.876±0.002	0.878±0.002	0.797±0.001
CAPRIN1	**0.8765**	0.858±0.012	0.860±0.009	0.843±0.003	0.827±0.002	0.745±0.008
DGCR8	**0.9236**	0.906±0.002	0.917±0.002	0.914±0.001	0.870±0.002	0.846±0.002
EIF4A3	**0.853**	0.799±0.003	0.823±0.004	0.812±0.001	0.820±0.001	0.717±0.005
EWSR1	**0.9443**	0.942±0.004	0.938±0.006	0.936±0.001	0.882±0.002	0.906±0.002
FMRP	0.8966	0.892±0.002	**0.900±0.002**	0.898±0.001	0.890±0.001	0.826±0.003
FOX2	**0.9618**	0.958±0.005	0.830±0.034	0.815±0.006	0.755±0.010	0.602±0.033
FUS	**0.8618**	0.855±0.004	0.859±0.002	0.858±0.002	0.799±0.003	0.770±0.003
FXR1	**0.9948**	0.994±0.001	0.959±0.009	0.952±0.003	0.871±0.003	0.942±0.004
FXR2	**0.9518**	0.939±0.009	0.941±0.003	0.938±0.002	0.868±0.002	0.896±0.004
HNRNPC	**0.9771**	**0.977±0.001**	0.976±0.001	0.972±0.000	0.973±0.001	0.970±0.001
HUR	0.8758	0.867±0.005	**0.879±0.006**	0.874±0.001	0.850±0.001	0.796±0.009
IGF2BP1	**0.8554**	0.843±0.002	0.845±0.003	0.843±0.001	0.835±0.003	0.760±0.009
IGF2BP2	**0.8426**	0.831±0.004	0.827±0.009	0.821±0.002	0.752±0.126	0.740±0.004
IGF2BP3	0.8229	0.816±0.004	**0.831±0.003**	0.822±0.002	0.754±0.122	0.706±0.003
LIN28A	**0.8751**	0.857±0.007	0.875±0.005	0.865±0.001	0.840±0.002	0.777±0.003
LIN28B	**0.9014**	0.892±0.004	0.889±0.005	0.882±0.001	0.758±0.129	0.822±0.003
METTL3	0.8649	0.852±0.009	**0.878±0.010**	0.854±0.003	0.808±0.003	0.772±0.007
MOV10	**0.8674**	0.838±0.006	0.845±0.005	0.849±0.001	0.778±0.004	0.777±0.008
PTB	**0.8347**	0.822±0.006	0.829±0.004	0.826±0.001	0.692±0.157	0.738±0.007
PUM2	**0.9758**	0.970±0.004	0.952±0.004	0.953±0.001	0.936±0.001	0.932±0.002
QKI	**0.9879**	0.971±0.006	0.927±0.005	0.921±0.003	0.866±0.004	0.866±0.007
SFRS1	**0.9684**	0.964±0.000	0.965±0.003	0.964±0.001	0.963±0.001	0.926±0.003
TAF15	**0.9945**	0.992±0.002	0.967±0.002	0.965±0.001	0.941±0.002	0.968±0.002
TDP43	**0.9336**	0.926±0.002	0.934±0.002	0.930±0.001	0.923±0.001	0.896±0.003
TIA1	**0.9666**	0.961±0.004	0.935±0.006	0.932±0.003	0.915±0.009	0.901±003
TIAL1	**0.9249**	0.917±0.003	0.906±0.003	0.902±0.001	0.898±0.002	0.871±0.005
TNRC6	**0.9797**	0.967±0.002	0.785±0.010	0.741±0.007	0.729±0.010	0.662±0.015
U2AF65	**0.9306**	0.926±0.002	0.930±0.002	0.928±0.001	0.911±0.001	0.899±0.004
WTAP	**0.9713**	0.967±0.002	0.794±0.069	0.793±0.011	0.808±0.022	0.732±0.009
ZC3HB	**0.8151**	0.804±0.003	0.804±0.005	0.792±0.002	0.794±0.004	0.697±0.008
AVG	**0.9188**	0.908±0.06	0.884±0.06	0.876±0.07	0.842±0.07	0.809±0.010

As shown in Table 1, the average values of CRBPDL, iCircRBP-DHN, PASSION, CRIP, CSCRites and CircSLNN are 0.9188, 0.908±0.06, 0.884±0.06, 0.876±0.07, 0.842±0.07 and 0.809±0.010, respectively. Obviously, our model improves the state-of-the-art performance in 28 of the 37 and accomplishes the supreme average AUCs of 0.9174, specifically in AGO1, AGO2, ALKBH5 and MOV10. And we provide the ROC curve of CRBPDL, and the average ROC curve (Fig 4C). The results fully indicate the enhancement of CRBPDL. At the same time, we also noticed that on the 6 data sets, the performance of CRBPDL has a very small gap with iCircRBP-DHN and PASSION, especially 4 of them are slightly worse than PASSION. The underlying reason may be that PASSION has extracted 6 types. This shows that manual features including richer sequence information can be used, and integrated optimization algorithms can also be used. In addition, CRBPDL is better than CircSLNN, but CircSLNN is a sequence tagging method that can predict the location of the binding site. Therefore, as a new research direction, we can consider whether we can improve the accuracy of predicting the position of the binding site, not just as a binary classification problem.

### Prediction performance of CRBPDL on 31 linear data sets

Similar to CRIP and PASSION, our CRBPDL also has the ability to identify linear RNA-protein interactions. To demonstrate the performance of our model CRBPDL, we compare it with ICIRCRBP-DHN, CRIP, iDeepS, DEEPBbind, CSCrites, and CIRCSLNN. To make a fair comparison, we used the same experimental data as the iCircRBP-DHN, and the results of the other comparative experiments were obtained directly from the literature [37]. The experimental results are shown in Table 2. From Table 2, CRBPDL obtained an average AUC of 0.9163, which is significantly superior than 0.895, 0.860, 0.842, 0.839, 0.833 and 0.803 of other methods. And on the 31 data sets, only the AUC of hnRNPC-1 is slightly lower than PASSION. In the remaining 30 data sets, our performance is still better than other methods. In addition, we have given the ACC on 31 data sets (Fig 4D), and we can find that the accuracy on the 31 data sets can meet the identification requirements of linear RNA-RBP binding sites.

**Table 2:** Comparison of prediction performance under different classification models on 31 linear RNA datasets.

dataset	CRBPDL	iCircRBP-DHN	CRIP	iDeepS	DeepBind	CSCRites	CircSLNN
AGO1234	**0.8178**	0.788±0.041	0.737±0.005	0.708	0.687	0.708±0.004	0.662±0.015
AGO2MNAS	**0.7995**	0.736±0.069	0.598±0.009	0.564	0.535	0.583±0.005	0.557±0.007
2-bingding_1	**0.9353**	0.925±0.011	0.862±0.004	0.788	0.811	0.842±0.003	0.795±0.006
2-bingding_2	**0.9439**	0.929±0.014	0.852±0.007	0.831	0.814	0.828±0.003	0.754±0.004
AGO2	**0.9088**	0.800±0.010	0.638±0.006	0.639	0.585	0.636±0.005	0.562±0.017
eIF4AIII_1	**0.9753**	0.963±0.004	0.952±0.001	0.942	0.925	0.937±0.004	0.894±0.005
eIF4AIII_2	**0.9851**	0.963±0.006	0.954±0.001	0.945	0.933	0.944±0.002	0.897±0.006
ELVAL1-1	**0.9482**	0.939±0.006	0.918±0.002	0.914	0.903	0.910±0.001	0.882±0.005
ELVAL1-MNASE	**0.7325**	0.695±0.050	0.604±0.007	0.567	0.546	0.581±0.010	0.520±0.13
ELVAL1-A	**0.9377**	0.922±0.006	0.898±0.002	0.888	0.869	0.876±0.002	0.845±0.006
ELVAL1-2	**0.9482**	0.943±0.002	0.926±0.001	0.937	0.914	0.925±0.001	0.898±0.002
EWSR1	**0.9217**	0.918±0.005	0.912±0.003	0.919	0.882	0.884±0.001	0.851±.004
FUS	**0.9596**	0.947±0.007	0.941±0.001	0.934	0.926	0.907±0.002	0.905±0.007
mut-FUS	**0.9621**	0.946±0.006	0.939±0.001	0.938	0.915	0.907±0.002	0.9070.013
IGF2BP1-3	**0.8156**	0.781±0.031	0.693±0.005	0.691	0.685	0.703±0.005	0.597±0.013
hnRNPC-1	0.9514	0.952±0.009	**0.963±0.001**	0.966	0.954	0.936±0.004	0.935±0.004
hnRNPC-2	**0.9891**	0.974±0.002	0.985±0.000	0.982	0.976	0.967±0.002	0.962±0.001
hnRNPL-1	**0.8583**	0.829±0.032	0.748±0.005	0.659	0.761	0.650±0.007	0.670±0.011
hnRNPL-2	**0.7995**	0.761±0.027	0.740±0.007	0.671	0.74	0.636±0.004	0.654±0.014
HnRNPL-like	**0.8008**	0.779±0.021	0.685±0.010	0.644	0.708	0.632±0.010	0.636±0.014
MOV10	**0.8971**	0.885±0.010	0.814±0.002	0.807	0.803	0.803±0.003	0.764±0.010
NSUN2	**0.8681**	0.832±0.008	0.865±0.003	0.789	0.847	0.798±0.004	0.776±0.015
PUM2	**0.9782**	0.969±0.003	0.963±0.003	0.966	0.937	0.959±0.002	0.920±0.004
QKI	**0.9786**	0.962±0.002	0.967±0.001	0.972	0.955	0.956±0.001	0.929±0.004
SFRS1	**0.9236**	0.912±0.007	0.886±0.004	0.888	0.86	0.885±0.003	0.794±0.008
TAF1S	**0.9336**	0.971±0.002	0.963±0.001	0.961	0.956	0.922±0.004	0.925±0.002
TDP-43	**0.946**	0.928±0.013	0.911±0.002	0.914	0.902	0.913±0.003	0.841±0.009
TIA1	**0.9636**	0.945±0.011	0.930±0.001	0.916	0.908	0.891±0.001	0.894±0.005
TIAL1	**0.9799**	0.915±0.012	0.898±0.002	0.885	0.881	0.864±0.004	0.847±0.009
U2AF65	**0.9782**	0.971±0.007	0.968±0.001	0.965	0.959	0.918±0.002	0.932±0.003
Y2AF65	**0.969**	0.951±0.005	0.935±0.002	0.927	0.916	0.906±0.004	0.893±0.002
Ave	**0.9163**	0.895±0.08	0.860±0.012	0.842	0.839	0.833±0.012	0.803±0.013

## Conclusion

In this paper, we design a new deep learning method, called CRBPDL, for circular RNA-RBP interaction site identification. Based on the MSRN framework, CRBPDL first connects the five codes into a single feature vector. Then MSRB is used to automatically explore higher-level local or global context dependencies and obtain high-level sequence features. Subsequently, the output of each MSRB is combined for global hierarchical feature fusion. And add self-Attention to grasp more critical and relevant features and improve prediction performance. Finally, an integrated deep learning network is constructed based on the Adaboost algorithm. Through the visualization of feature representation, this unique architecture has proven to be effective. To verify CRBPDL, we performed predictions of the binding sites of circRNA and linear RNA and evaluated the performance of different methods. The comparison of 37 circular RNA data sets and 31 linear RNAs not only proves the effectiveness of our method but also shows the potential of the model in the identification of circular RNA-RBP interaction sites. Currently, there are few data on known RBP binding sites. The positive and negative samples are unbalanced. Therefore, the most important thing is that future research is to expand the data set, collect RBP binding sites that bind to circRNA, lncRNA or other RNAs, explore their binding characteristics, and develop universal prediction software.

## Materials and methods

### Data sets

To prove the effectiveness of our proposed CRBPDL and make a fair comparison with other tools at the same time, we used the benchmark data set (named as ‘circRNA_RBP-37’) used in [35,37,42]. The data set consists of 37 RBPs downloaded from the circinteractome database (https://circinteractome.nia.nih.gov/) [19]. The database collects RBP bound to mature circular RNA and RBP bound to the upstream and downstream sequences of mature circular RNA. Since RBP binding may play a role in regulating splicing events near the splicing site, we considered all RBP binding sites in this study. In the end, we obtained a total of 32,216 circular RNAs related to 37 circular RNA data sets. Among them, the positive sample came from the interaction site on the circular RNA verified by the laboratory. In each CLIP-seq peak, the sequence fragment with a length of 101 nucleotides (nt) was centered and extends 50 nucleotides (nt) in both directions. At the same time, negative sequences were randomly selected from the left-over circular RNA fragments. Subsequently, we applied the same postprocessing method to extract the 101 nucleotide length (nt) binding sites/residual intermediate readings in the previous work [17,18]. Since sequence similarity will influence the consistency of the ML, we used CD-HIT to eliminate the sequence with a similarity threshold of 0.8, which is the same as in CRIP and PASSION. After removing sequence redundancy, we got the final data set, namely the positive and negative samples are 335,976 and 335,976 respectively. 80% of the data sets were selected as training set, and 20% were used as test set.

Additionally, refer to other studies [17,18], we compared the efficiency of CRBPDL to identify the linear RNA -RBP interaction sites. We downloaded the linear RNA data set from PASSION [35] and iDeepS [22], which includes the linear RNA dataset of CLIP-Seq data combined with 31 RBP. Each data set has 5,000 training sets and 1,000 test sets.

### Feature encoding

#### k-nucleotide frequencies

To characterize the local context features of circular RNA sequences, we used KNF coding sequences. KNF describes the frequency of all possible polynucleotides of k nucleotides in the sequence. In this study, we took k = 1, 2, 3, namely single-nucleotide composition frequency, dinucleotide composition frequency and trinucleotide composition frequency. KNF retains a large number of original sequence patterns and integrates a variety of sequence information [43,44]. Compared with traditional single hot spot representation [45], KNF effectively compensates for the lack of information.

#### Doc2vec

In recent DL model research, to learn more sequence context and semantic information, an increasing number of sequence studies have adopted continuous, high-dimensional word embedding-based coding to substitute one-hot coding, and have achieved good results. Therefore, based on the circRNA corpus of circBase [46], we used the Distributed Memory Model of Paragraph Vectors (PV-DM) model of the Doc2Vec algorithm to vectorize the sequence [47] and train the vectorized model Doc2Vec.model. After that, sequence data were input into the model, 10-mer sequence fragments were taken as circular RNA words, and word embedding training was used to obtain feature vectors. In this way, learning as a continuous distribution representation of global context features expands the vocabulary and can capture the semantics and grammar in these subsequences for long-term dependency modeling.

#### Electron–ion interaction pseudopotential

The EIIP [48] describes the characteristics of free electron energy on the circRNA sequences. EIIP was widely used to predict the binding sites of RBPs. The EIIP values of the four characters that may appear in the sequence (ie, “A”, “T”, “C”, “G”) are 0.1260, 0.1335, 0.1340 and 0.0806. Hence the EIIP coding method can be used to encode DNA sequence as a digital vector. For example, AATCCGA encoding is a numeric vector consisting of (0.1260, 0.1260,0.1335, 0.1340, 0.1340, 0.0806,0.1260).

#### Chemical characteristic of nucleotide

Each nucleotide has three types of chemical characteristics (CCN): chemical functions (including amino and keto groups), ring structure (including bicyclic purines and monocyclic pyrimidines), and hydrogen bonds (including weak hydrogen bonds and strong hydrogen bonds) [49]. For the ring structure, A and G belong to purines, coded as 1, and C and T belong to pyrimidines, coded as 0. For chemical functions, A and C belong to amino groups, coded as 1; G and T belong to ketone groups, coded as 0. For hydrogen bonds, A and T belong to a weak hydrogen bond, coded as 1, while C and G belong to a strong hydrogen bond, coded as 0. For example, AATCCGA can be encoded as (1,1,1,1,1,1,0,0,1,0,1,0,0,1,0,1,0,0,1,1,1).

#### Accumulated nucleotide frequency

ANF presents the density characteristics of nucleotide sequence [49]. Suppose a circRNA sequence S = s_1_s_2_…s_i_, where i is the length of S. S_j_ = s_1_s_2_…s_j_, j is the length of S_j_. S_j_ is the j-th prefix sequence of S. Then the ANF calculation formula is:

$$ANFs_{j}=\frac{f(s_{j})}{j}$$ (1)

$$f(s_{j})=\sum_{n=1}^{j}T(s_{t}),T(s_{t})=\left\{ \begin{array}{c} 1,s_{t}=s_{j}\\ 0,s_{t}\neq s_{j} \end{array}\right.$$ (2)

### Multiscale residual network

To obtain rich feature information, a multi-scale CNN layer is constructed to capture high-level features. Unlike traditional Convolutional Neural Networks, different from traditional CNN, multiscale residual network can improve the information trend flow and gradient of the whole network, reduce the computational complexity and improve the model performance [50].

In the MSRN framework, due to the different distributions of the five feature descriptors, we employed convolution filters on five characteristics with a convolution kernel of 128 and then cascaded, which is a common method to balance the distribution of biological features. Afterwards, the MSRN framework contained a shallow CNN extraction layer, and the size of the convolution kernel was 3. Then, the inception module, including 6 cascaded multiscale residual block (MSRB) modules, was used, and the convolution kernel was 64. Each MSRB includes a 3 convolutional layer. Based on the hierarchical feature fusion structure (HFFS), the output of each MSRB was combined to perform global feature fusion. Subsequently, following input to a layer of convolution kernel, there were 192 filters, and a 1×1 convolution can increase and decrease the number of channels, organize information across channels, and increase feature transformation with a small amount of calculation and nonlinear transformation to improve the network expression ability. After that, there was a merge layer with a dropout value of 0.4.

### bidirectional gating recurrent unit

For circRNA sequences, besides local background information, there are also long-chain dependencies [51]. Multiscale residual block network can capture only the dependencies between sequences. Therefore, the study employed a Bidirectional GRU to obtain context information from the front and back at the same time to improve the performance.

Bidirectional GRU has only two gates, namely, the update and the reset gate. The update gate controls the extent to which the state information at the previous moment is brought into the current state. The larger the value of the update gate, the more the state information at the previous moment is brought in. The reset gate is used to control the degree of ignoring the state information at the previous moment. The smaller the reset gate, the more information is ignored. The bidirectional GRU can adaptively change its state according to the input, thereby solving the problem of vanishing gradient in RNN.

### Self-Attention

The self-attention mechanism was to adaptively pay attention to and learn an important part according to the needs, and ignore the insignificant part. It was widely used in various deep learning applications, including vision processing, phosphorylation site prediction, drug target prediction, etc. [52]. The intention of the attention mechanism is to neglect insignificant word in the bulk of information, selectively filter out a particle of important information, and and express the importance of the information by calculating the weight of the information.

In this research, in our model CRBPDL, the output matrix of the BiGRU layer and its transposed matrix were input into the attention layer, and different features were given different weights, and important features were selected from the dimensional features. Abandon some secondary features and used sigmoid as the activation function.

### Implementation

CRBPDL was implemented using the Keras 1.1.2 library in Python. First, we used 80% of the benchmark data set for the training and 20% for testing. Then, on the training set, 80% for training and 20% for verification. Acc was used to evaluate each parameter setting. The verification data set was applicable to monitor the astringency of each stage in the training process, and the training process can be quitted in advance. The study adopted the update method of the learning rate of the Adam gradient descent algorithm, where the initial learning rate is set to 0.001, the max epochs is 200, the epochs is 30, and the batch size is 50. In addition, we have also adopted a variety of techniques to prevent or reduce overfitting, such as batch normalization [50], dropout [51] and early stopping. We used the selected optimal parameter settings, used all training data to train the model, chose the model with the greatest performance as the base model, employed AdaBoost for ensemble, and applied the integrated model as the computational model. AdaBoost is an iterative algorithm. Its core idea is to train different classifiers (weak classifiers) for the same training set, and then group these weak classifiers to form a stronger final classifier (strong classifier) [53,54].

### Evaluation metrics

In this study, we employed five evaluation metrics: sensitivity (SE), specificity (SP), accuracy (ACC), Matthew’s correlation coefficient (MCC) and AUC [55–63], defined as follows:

$$SE=\frac{TP}{TP+FN}$$ (3)

$$SP=\frac{TN}{TN+FP}$$ (4)

$$ACC=\frac{TN+TP}{TN+FP+TP+FN}$$ (5)

$$\mathrm{MCC}=\frac{\left(TP\times TN)-(FP\times FN\right)}{\sqrt{\left(TP+FP)\times (TP+FN)\times (TN+FP)\times (TN+FN\right)}}$$ (6)

where TP, TN, FP and FN denote the numbers of true positives, true negatives, false positives and false negatives, respectively. Furthermore, the area under the curve (AUC) is the area under the ROC curve.

## Supporting information

S1 Text Model performance analysis under different EPOCH. (DOCX)
