# Supplementary material for: CRBPDL: Identification of circRNA-RBP interaction sites using an ensemble neural network approach
Source: PLoS Comput Biol. 2022 Jan 20;18(1):e1009798. doi: 10.1371/journal.pcbi.1009798 (PMC8806072; doi:10.1371/journal.pcbi.1009798)
Supplement: S1 Text — (DOCX) [file pcbi.1009798.s001.docx]

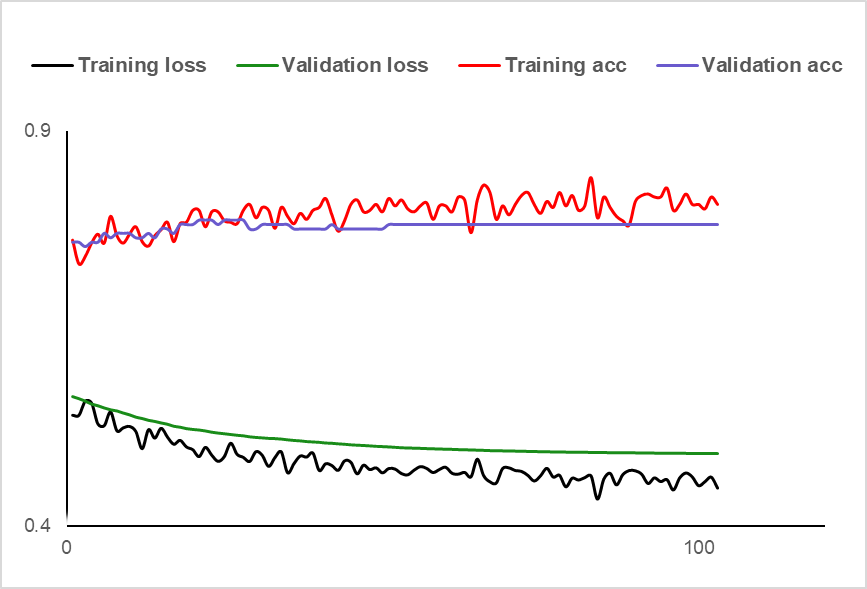


(a) AGO3 (b) ALKBH5

(c) AUF1 (d) C17ORF85

(e) C22ORF28 (f) CAPRIN

(g) EWSR1 (h) FUS

(i) FXR1 (j) FXR2

(k) HNRNPC (l) HUR

(m) IGF2BP1 (n) IGF2BP2

(o) IGF2BP3 (p) LIN28A

(q) LIN28B (r) METTL3

(s) MOV10 (t) PTB

(u) PUM2 (v) QKI

(w) SFRS1 (x) TAF15

(y) TDP43 (z) TIA1

(a1) TIAL1 (b1) TNRC6

S1 Text. Model performance analysis under different EPOCH.
